# Editorial: Advances in Primary Immunodeficiencies in India

**DOI:** 10.3389/fimmu.2021.701335

**Published:** 2021-06-24

**Authors:** Sudhir Gupta, Shobha Sehgal

**Affiliations:** ^1^ Division of Basic and Clinical Immunology, University of California at Irvine, Irvine, CA, United States; ^2^ Department of Immunopathology, Postgraduate Institute for Medical Education and Research, Chandigarh, India

**Keywords:** prenatal diagnosis, SCID, XLA, WAS, LAD, MSMD, HSCT, FLH

Primary immunodeficiency diseases (PIDs) or inborn errors of immunity (IEI) are an ever-expanding universe of genetic defects of the immune system. In this editorial, we will be using PIDs and IEI interchangeably. In the last almost 70 years, since X-linked agammaglobulinemia (Bruton’s agammaglobulinemia) was discovered by Col. Ogden Bruton, more than 460 IEI have been described and more than 400 mutated genes have been assigned to these disorders. This has been primarily due to advances in cellular and molecular technologies, including NGS, that are used to perform WES and GWS. We began with four categories of PIDs (Combined immune deficiency, Antibody deficiency, T cell deficiency, and Complement deficiency), and now IEI are classified into 10 different categories.

In India, the first description of PID was reported in two siblings with ataxia telangiectasia in 1975 exclusively on clinical presentation (1). Since then and until 2010, the majority of PIDs were described on clinical criteria and very little laboratory confirmation. In 2010, Sudhir Gupta established the Foundation for Primary Immunodeficiency Diseases (FPID; www.fpid.org) to improve awareness, provide education and training, and support treatment and research for PIDs in India. Currently, there are eight regional FPID diagnostic and Treatment Centers at eight different premier medical institutions spread all over India. Two of these centers are now designated as Center for Excellence in PIDs that are fully equipped with the capability of molecular diagnoses. In addition, there are several commercial laboratories, where gene mutation analyses are available. Increased awareness and availability of molecular diagnoses have resulted in increased referrals of patients suspected of IEI and therefore increased and early diagnosis. In the last 10 years, the landscape of IEI in India has changed dramatically. As recently as 10 years back, there were only two centers that were performing HSCT for PIDs. Currently, there are more than 15 center swhere HSCT for PIDs are actively performed. More than 250 scientific papers on IEI have been published from FPID centers. This special Research Article on “Advances in Primary Immunodeficiency Diseases in India” is a compilation of 11 articles presenting data on 1,539 patients with IEI and highlighting the progress made in the last decade in the diagnosis and treatment of PIDs. The 10 most common IEIs have been presented. Furthermore, the authors of these articles also emphasized financial, cultural, and institutional challenges that limit the diagnosis and therapy of patients with IEI.

India, unlike the US and some other Western countries, does not have a national newborn screening program. However, they are using prenatal diagnosis (PND) for IEI. Yadav et al. from the Madkaikar group discussed the PND services available and the economic, ethical, and cultural challenges associated with genetic counseling. Mutation detection in the index case and analysis of chorionic villous sampling or amniocentesis are the preferred procedures for PND and phenotypic analysis of cordocentesis sample is reserved for families with well-characterized index case seeking PND in the latter part of the second trimester of pregnancy. Of 112 families investigated for PID, PID was confirmed in 32 families. These included diagnoses of SCID, LAD, FHL, and CGD. Therefore, in families having a child affected with PID, genetic counseling and PND are the cornerstones of primary preventive management. Prenatal diagnosis provides a choice for the family to carry on or terminate the pregnancy. Needless to say, there are ethical issues involved.

There has been an exponential rise in the diagnosis of SCID. Vignesh et al. presented data on 254 patients with SCID; the molecular diagnosis was made in 192 patients, and, in the order of frequency, the mutations in genes included IL-2RG, RAG1, ADA, RAG2, JAK3, DCLRE1C, *IL7RA*, *PNP*, *RFXAP*, *CIITA*, *RFXANK*, *NHEJ1*, *CD3E*, *CD3D*, *RFX5*, *ZAP70*, *STK4*, *CORO1A*, *STIM1*, *PRKDC*, *AK2*, *DOCK2*, and *SP100*.

Rawat et al. presented a clinical and genetic profile of 137 patients with an established diagnosis of X-linked agammaglobulinemia. Missense variants in the *BTK* gene were the most common, followed by frameshift and nonsense variants. Most pathogenic gene variants were clustered in the distal part of the gene encompassing exons 14–19 encoding for the tyrosine kinase domain. There were delays in diagnosis because of the lack of availability of facilities for molecular diagnoses at several centers.

Missense variants are most commonly observed in patients with Wiskott Aldrich syndrome. Suri et al. presented clinical and molecular data from 81 patients with Wiskott-Aldrich syndrome (WAS). They reported 24 novel variants, most of these being frameshift and nonsense mutations resulting in premature termination of protein synthesis.

Saikia et al. reported seven novel STAT3 variants, including a rare linker domain nonsense variant and a CC domain variant in patients with Hyper IgE syndrome. Not surprisingly, because of endemic mycobacterial diseases in India, *Mycobacterial* diseases in HIES were more frequent compared to the Western world.

Clinical and molecular data on a large cohort of patients with chronic granulomatous disease (CGD) are presented (Rawat et al.). The proportion of patients with AR-CGD is higher as compared to Western cohorts; however, regional differences within India were observed. *CYBA* variants were documented only in Southern and Western parts of India; a common dinucleotide deletion in *NCF2* (c.835_836delAC) was noted only in the North Indian population.

Kambli et al. presented clinical and molecular data on a large cohort of 132 patients with leukocyte adhesion deficiency (LAD). Around 96% of patients were affected with LAD-1 and none with LAD-2. A total of 30 novel mutations were detected in the *ITGβ2* gene, and 4 novel mutations were detected in the *FERMT3* gene.

Clinical data on a large group of molecular-defined familial hemophagocytic lymphocytosis (FHL), and IEI characterized by immune dysregulation are reviewed (Shabrish et al.). The majority of patients were FHL-2 and FHL-3. Molecular characterization of respective genes revealed 76 different disease-causing mutations, including 51% novel mutations in the *PRF1, UNC13D, STX11*, and *STXBP2* genes.

Almost all types of autoinflammatory diseases are represented in the Indian population. Suri et al. presented clinical and molecular data on a large cohort of autoinflammatory disorders that included type 1 interferonopathies, diseases affecting inflammasomes, with non-inflammasome related conditions periodic fever, aphthous stomatitis, pharyngitis, and adenitis (PFAPA). Type1 interferonopathies identified in the cohort included patients with deficiency of adenosine deaminase 2 (*DADA2)*, STING-associated vasculopathy with onset in infancy (SAVI), and spondyloenchondro-dysplasia with immune dysregulation (SPENCD). Diseases affecting inflammasomes included mevalonate kinase deficiency, cryopyrin-associated periodic syndromes (CAPS), NLRP12, familial mediterranean fever (FMF), autoinflammation, PLCG_2_-associated antibody deficiency, and immune dysregulation (APLAID), TNF receptor-associated periodic syndrome (TRAPS), A20 haploinsufficiency, and deficiency of interleukin 1 receptor antagonist (DIRA).

Because of the practice of universal BCG vaccination, disseminated BCGosis is a common manifestation in certain PIDs. Taur et al. described BCGosis in more than 80% of patients with MSMD involving abnormalities of the IL-12/IL-23/ISG15/IFN-γ axis. Authors went on to suggest that all patients with BCGosis and suspected of MSMD, irrespective of age, should be investigated for abnormality of the IL-12/IL-23/ISG15/IFN-γ circuit.

During the last decade, the number of centers performing HSCT for PIDs has increased, and, therefore, the number of PID patients undergoing HSCT has exponentially increased. Raj et al. presented data on HSCT in 228 children with PIDs. The most common PIDs undergoing HSCT were SCID (25%) and HLH (25%). Others included WAS and CGD. In the last 5 years, the survival of children with PID undergoing HSCT in India has improved. HSCT protocols have been modified to reduce the cost without compromising survival that included doing away with T cell depletion.

India with a population of 1.3 billion is expected to have more than 1 million patients with IEI. In this compilation, data for just more than 1360 patients have been presented. Therefore, the prevalence of IEI in India is not known. However, with recent approval and funding of a national PID registry by the Indian Counsel for Medical Research, it should be possible. Although unprecedented advancements have been made in the diagnosis and treatment of patients with IEI in a short span of 10 years, a number of economical, ethical, and cultural challenges remain. India will need to continue to increase awareness, support education, training, and technology that would result in early and improved diagnosis and successful treatment. However, this would require a concerted effort between the Government of India, the State government, institutions, and NGOs. Recently, the Government of India in their National Rare Disease Policy has included PIDs. FPID is committed to continuing to provide necessary academic, technical, political, and financial support for those with IEI.

## Author Contributions

SG wrote major component of the Editorial. SS provided critical historical data of publications since 1975. All authors contributed to the article and approved the submitted version.

## Conflict of Interest

The authors declare that the research was conducted in the absence of any commercial or financial relationships that could be construed as a potential conflict of interest.
